# Association between Severity of Diabetic Retinopathy and Cardiac Function in Patients with Type 2 Diabetes

**DOI:** 10.1155/2023/6588932

**Published:** 2023-06-07

**Authors:** YanYan Chen, MengYing Li, Yi Wang, JianFang Fu, XiangYang Liu, Ying Zhang, LiWen Liu, ShengJun Ta, ZuoWei Lu, ZePing Li, Jie Zhou, XiaoMiao Li

**Affiliations:** ^1^Department of Endocrinology, Xijing Hospital, Air Force Medical University, Xi'an, Shaanxi 710032, China; ^2^Department of Ultrasound, Xijing Hospital, Air Force Medical University, Xi'an, Shaanxi 710032, China; ^3^Nanchang University Queen Mary School, Nanchang 330038, China

## Abstract

**Background:**

The purpose of this research was to assess the relationship between the severity of diabetic retinopathy (DR) and indexes of left ventricle (LV) structure and function in type 2 diabetes mellitus (T2DM).

**Methods:**

Retrospective analysis of 790 patients with T2DM and preserved LV ejection fraction. Retinopathy stages were classified as no DR, early nonproliferative DR, moderate to severe nonproliferative DR, or proliferative DR. The electrocardiogram was used to assess myocardial conduction function. Echocardiography was used to evaluate myocardial structure and function.

**Results:**

Patients were divided into three groups based on the DR status: no DR group (NDR, *n* = 475), nonproliferative DR group (NPDR, *n* = 247), and proliferative DR group (PDR, *n* = 68). LV interventricular septal thickness (IVST) increased significantly with more severe retinopathy (NDR: 10.00 ± 1.09; NPDR: 10.42 ± 1.21; and PDR: 10.66 ± 1.58; *P* < 0.001). Multivariate logistic regression analysis showed that the significant correlation of IVST persisted between subjects with no retinopathy and proliferative DR (odds ratio = 1.35, *P* = 0.026). Indices of myocardial conduction function were assessed by electrocardiogram differences among groups of retinopathy (all *P* < 0.001). In multiple-adjusted linear regression analyses, the increasing degree of retinopathy was closely correlated with heart rate (*β* = 1.593, *P* = 0.027), PR interval (*β* = 4.666, *P* = 0.001), and QTc interval (*β* = 8.807, *P* = 0.005).

**Conclusion:**

The proliferative DR was independently associated with worse cardiac structure and function by echocardiography. Furthermore, the severity of retinopathy significantly correlated with abnormalities of the electrocardiogram in patients with T2DM.

## 1. Introduction

Cardiovascular disease (CVD) is a leading cause of death in patients with type 2 diabetes mellitus (T2DM) [1]. As one of the chronic complications of diabetes, the clinical pathologic entity has been proposed for “diabetic cardiomyopathy” (DCM), featured by the presence of myocardial dysfunction without proof of coronary artery disease [2]. Recently, the increased occurrence of cardiovascular events has been attributed to DCM [3, 4], and diastolic dysfunction is regarded as the first functional change in the course of DCM [5, 6]. In addition, an increase in the risk of death caused by T2DM, not only from CVD but also from the sudden death of the heart [7], is associated with myocardial abnormalities and electrical propagation within the heart. Diabetes-related microvascular dysfunction is considered to be one of the suggested pathologies contributing to adverse alterations in the myocardium [8]. The “common soil” theory of diabetic complications has been reported and suggests that the molecular mechanisms of diabetes share a common pathway to microvascular complications [9].

Diabetic retinopathy (DR), as a representative of the potential for microvascular impairment, is also known to predict future cardiovascular events among T2DM [10, 11]. Based on a worldwide epidemiological survey, the study indicates that 35% of people with diabetes had some form of retinopathy and that 7% had proliferative retinopathy [12]. Therefore, it is critical to make efforts to fully understand the association between the increased burden of proliferative retinopathy and the occurrence of heart failure in patients with diabetes who are at a high risk of CVD morbidity and mortality. Some investigators have reported that more restricted retinal artery vessel diameter was related to left ventricle (LV) concentric reshaping in asymptomatic individuals with diabetes [13], implying that microvascular disorders might not only facilitate cardiac restructure but also diabetes-related arrhythmia.

This interaction between DR and myocardial damage is part of a wider debate on the correlation between micro- and macrovascular diabetic complications, which is not only documented by important prospective observational studies [14] but also very recently by intervention studies [15]. However, these studies pay more attention to the myocardial dysfunction between patients with and without DR. Under the theory that structure determines function, they have not specifically taken the severe nature of retinopathy and cardiac structure into account. In addition, though some studies have been reported, the data still has to be interpreted with caution given the small sample size, and the impact of the complex advances in electrical propagation abnormalities on DR has not been fully elucidated. Thus, we performed a relatively large sample clinical study to investigate the relationship between the severity of retinopathy and the structure and function of the heart in patients with T2DM.

## 2. Method

### 2.1. Samples

**Figure 1:**
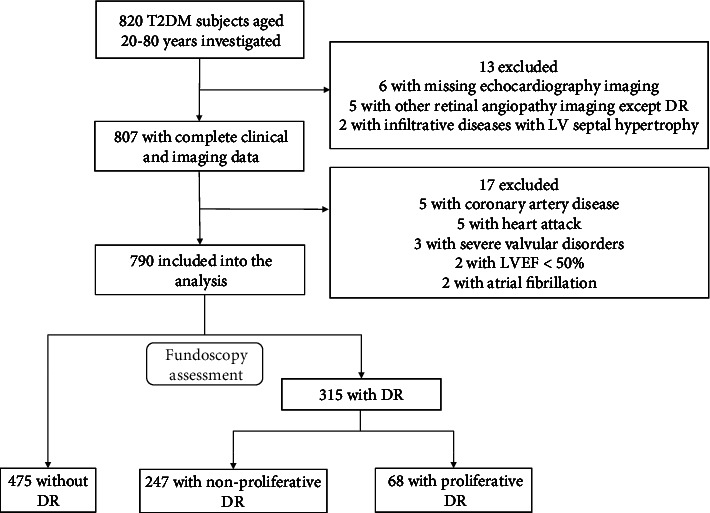
Flow chart for the selection of the study subjects. T2DM: type 2 diabetes mellitus; DR: diabetic retinopathy; LVEF: left ventricular (LV) ejection fraction.

It was a retrospective study performed in the Department of Endocrinology of Xijing Hospital of Air Force Medical University. A total of 820 patients with T2DM who visited the hospital from March 2013 to March 2016 were enrolled. The present study protocol was reviewed and approved by the Institutional Review Board of Xijing Hospital (Approval No. XJLL-KY20222107). Informed consent was submitted by all subjects when they were included. Based on their clinical status, previous medical documents, electrocardiogram at baseline, and routine blood screenings, we excluded patients with (1) no records of fundoscopy and echocardiography findings, (2) any retinal angiopathy except DR, (3) infiltrative diseases with LV septal hypertrophy, (4) coronary artery disease, (5) heart attack, (6) severe valvular disorders, (7) left ventricular ejection fraction (LVEF) < 50%, and (8) atrial fibrillation. The remaining 790 patients were admitted to this study (Figure 1).

### 2.2. Patient Information

Both demographic and clinical variables were derived from medical files: age, diabetes duration, gender, body mass index (BMI), glycosylated hemoglobin (HbA1c), estimated glomerular filtration rate (eGFR), uric acid, serum lipid profiling (e.g., triglyceride, total cholesterol, and high-density lipoprotein cholesterol (HDL-C)), diastolic blood pressure and systolic blood pressure, and medications including angiotensin converting enzyme inhibitor (ACEI)/angiotensin receptor blocker (ARB) and insulin use. Smoking history was recorded as yes if the patient was a present or previous smoker. Diabetic nephropathy was clinically diagnosed based on the reduced eGFR (<60 ml/min/1.73 m^2^) and/or micro-/macroalbuminuria (>30 mg/l) revealed by two measurements performed 3 months apart, while excluding another chronic kidney disease by an experienced clinician.

### 2.3. Echocardiographic Assessment

All patients underwent a complete transthoracic echocardiographic examination using commercially available echocardiographic machines (Vingmed Vivid 7, General Electric Vingmed Ultrasound, Milwaukee, WI, USA) with the patient lying in the lateral decubitus position. Off-line analysis was performed using General Electric EchoPAC software (BT11). The LV dimension and interventricular septum thickness were measured in M-mode echocardiography. The acquired data consist of left atrial diameter, LV end-systolic dimension (LV ESD), interventricular septum thickness (IVST), LV fractional shortening, LV end-diastolic dimension (LV EDD), stroke volume, LV end-diastolic volume (LV EDV), and LV end-systolic volume (LV ESV). LVEF was determined using the modified Simpson's biplane method. The assessment of LV diastolic function was determined based on pulsed-wave Doppler of mitral inflow. Peak early diastolic (E-wave) and late diastolic (A-wave) velocities were recorded, and the E/A ratio was also calculated [16]. According to the previous recommendations, diastolic function was defined as abnormal for E/A ratios < 1 [17].

### 2.4. Diabetic Retinopathy Assessment

Retinopathy conditions were derived from fundoscopy performed by an ophthalmologist. Combined with fundus examination and the international DR grading standard in 2003 [18], retinopathy stages were classified as no retinopathy, early nonproliferative DR, moderate to severe nonproliferative DR, or proliferative DR. The bias was controlled by the double-blind method.

### 2.5. Electrocardiogram Assessment

Electrocardiogram parameters were obtained from the medical records. All subjects were analyzed and measured by standard 12-lead synchronous electrocardiogram by Japan photoelectric electrocardiogram machine. The interval lengths of PR, QRS, QT, and QTc were automatically analyzed to generate measurement data, including heart rate. The PR interval is the process from the beginning of the P wave to the QRS complex, and the normal range of adults lasted 120 ms to 200 ms; a PR interval > 200 ms is considered prolonged [19]. The QT interval is the process between the beginning of the QRS complex and the end of the T wave. The corrected QT interval (QTc) is based on Bazett's formula (QTc = QT/HRR), and the QTc > 440 ms is considered to be prolonged [20].

### 2.6. Statistical Analysis

The Shapiro-Wilk test was used to evaluate the Gaussian distribution, and if the assumption of normality was not met, the nonparametric test was used. Categorical variables were shown as percentages *n* (%) and continuous variables as means ± SD. The overall differences between multiple groups were analyzed by one-way analysis of variance (ANOVA). Post hoc multiple comparisons used the least significant difference (LSD) test when the ANOVA test was significant (*P* < 0.05). Categorical variables were compared by the contingency chi-square test. Correlations of the DR status with the parameters of electrocardiogram were examined using linear regression analysis, and they were given in the form of Pearson's correlation coefficients. By stepwise selection, we included variables with *P* values < 0.05 in the multivariate model, and no multicollinearity existed between variables by variance inflation factor (VIF) check [16]. The relationship between DR stage and echocardiographic parameters was analyzed by multinomial logistic regression in a univariate model and further adjusted 12 confounding factors for age, gender, diastolic and systolic blood pressure, hypertension status, total cholesterol, HDL-C, diabetic nephropathy, smoking history, diabetes duration, ACEI/ARB, and insulin use in the multivariate model, given the values of odds ratio (OR) and 95% confidence interval (CI). All statistical analyses were conducted by SPSS Statistics version 26.0. The *P* < 0.05 on both sides was considered statistically significant.

## 3. Results

### 3.1. Basic Features

Baseline patient characteristics based on retinopathy status are displayed in Table 1. A total of 790 patients (66.3% males, mean age 52.87 years old) were separated into three groups: no DR group (NDR, *n* = 475), nonproliferative DR group (NPDR, *n* = 247) including early nonproliferative retinopathy or moderate to severe nonproliferative retinopathy, and proliferative DR group (PDR, *n* = 68). There were no significant differences in BMI, triglyceride, uric acid, or HbA1c across groups of retinopathy. Patients with nonproliferative or proliferative retinopathy were primarily females, with longer duration of diabetes, older age, higher systolic and diastolic blood pressure, and lower eGFR; they had higher levels of total cholesterol and HDL-C and a higher prevalence of hypertension and diabetic nephropathy, compared to patients without retinopathy. Moreover, smoking history, insulin, and ACEI/ARB use were also more common in patients with retinopathy.

### 3.2. Echocardiographic Parameters

The Doppler echocardiographic data based on retinopathy status are displayed in Table 2. No difference in LVEF or fractional shortening was observed across groups of retinopathy. Cardiac parameters of LV structural changes (e.g., IVST, left atrial diameter, LV EDD, LV ESD, LV EDV, LV ESV, and stroke volume) were significantly different in patients with nonproliferative or proliferative retinopathy compared to patients without retinopathy (*P* < 0.01 for all). E/A ratio < 1 was found in 659 cases (84.6%) and 78.3%, 94.2%, and 94.1% of those with no retinopathy, nonproliferative retinopathy, and proliferative retinopathy, respectively. In addition, compared with those without retinopathy, the E/A ratio was significantly decreased in patients with nonproliferative or proliferative retinopathy (0.90 ± 0.32, 0.78 ± 0.19, and 0.76 ± 0.22, respectively; *P* < 0.001).

### 3.3. Electrocardiogram Analysis

Main electrocardiogram characteristics based on retinopathy status are displayed in Table 3. Patients with nonproliferative or proliferative retinopathy had higher heart rates and longer PR and QTc intervals; moreover, they had a higher prevalence of PR interval > 200 ms and QTc interval > 440 ms compared to those without retinopathy.  Table 4 illustrates a stepwise multiple linear regression analysis for clinical variables associated with the electrocardiogram parameters. As shown, even in the controlled 12 clinical confounding factors for age, gender, diastolic and systolic blood pressure, hypertension, total cholesterol, HDL cholesterol, diabetic nephropathy, smoking history, diabetes duration, ACEI/ARB, and insulin use in multiple regression analysis, the DR stage was still independently associated with heart rate (*β* = 1.593, *P* = 0.027), PR interval (*β* = 4.666, *P* = 0.001), and QTc interval (*β* = 8.807, *P* = 0.005) (Table 4).

### 3.4. Echocardiography Analysis

The associations between echocardiography and retinopathy were examined in univariable, age- and sex-adjusted, and multivariable logistic regression models in Tables 5 and 6. As shown in a univariable model, the main LV geometry parameters (e.g., IVST, left atrial diameter, LV EDV, and stroke volume) showed a significant correlation across groups of retinopathy (*P* < 0.05 vs. NDR, respectively) (Table 5). Likewise, diastolic function indicators (e.g., A velocity, E/A ratio, and E/A ratio < 1) showed the same result (*P* < 0.01 vs. NDR, respectively). However, such an association was not found with LVEF, LV fractional shortening, or E velocity between groups. After adjusting age and sex, echocardiography parameters (IVST, left atrial diameter, A velocity, and E/A ratio) were independently associated with the presence of proliferative retinopathy (Table 5). In the multivariate model, further adjusted for the 12 possible confounders mentioned above, echocardiography parameters (LV EDD, LV ESD, EDV, and ESV) were still independently associated with the presence of nonproliferative retinopathy. However, for the presence of proliferative retinopathy, most of the echocardiography parameters were not associated with it. Interestingly, the independent association between higher IVST and proliferative retinopathy remained persistent (OR, 1.35; 95% CI, 1.04 to 1.75; *P* = 0.026 for IVST) (Table 6).

## 4. Discussion

The present study examined the relationship between the severity of retinopathy and indices of cardiac structure and function evaluated by conventional echocardiography and electrocardiogram in patients with T2DM. Our findings demonstrated that worsening indices of cardiac structure and function were significantly correlated with more severe retinopathy in asymptomatic T2DM patients with preserved LVEF.

### 4.1. Retinopathy and Cardiac Microangiopathy

As we know, DR is the most characteristic microvascular complication of diabetes. It is commonly used as a substitute marker for diabetic microangiopathy in numerous clinical studies. Meanwhile, its presence may indicate microcirculation dysfunction in other organs [21]. As the saying goes, the eyes are the windows of the heart. Thus, several clinical studies have also linked DR to the heart. For instance, Akasaka et al. [22] observed that the coronary flow reserve was strongly limited in patients with diabetes mellitus and was more pronounced in those with advanced retinopathy. Additionally, a study using PET/CT has also confirmed that the incidence of coronary microvascular impairment is much higher among people with type 2 diabetes without overt coronary artery disease, especially with concomitant albuminuria, further supporting the concept of common microvascular injury occurring in multiple microvascular beds [23]. Others further reported that cardiac microangiopathy is characterized by thickening of the capillary basement membrane, microvascular spasm, and capillary microaneurysms [8]. It is not surprising that those similar background pathogeneses are also present in DR, and the more severe the retinopathy, the thicker the choroid [24]. Overall, DR is strongly associated with cardiac microangiopathy. Its presence may have extra adverse effects on myocardial function and could lead to an increased incidence of heart failure [25, 26]. Based on these findings, our study further suggests that DR and microvascular heart disease are not completely separate. Microangiopathy may play an essential pathogenic role in developing cardiac dysfunction, or a common fundamental pathway exists among these disorders [27].

### 4.2. Retinopathy and Echocardiography

The E/A ratio has previously been shown to be an indicator of ventricular diastolic dysfunction [28, 29]. Our results found that the more severe the retinopathy, the lower the E/A ratio, further supporting the evidence that DR has correlated with LV diastolic impairment [16, 30]. In addition, we also focused on the association between DR and myocardial structure. Surprisingly, we found that LV remodeling was strongly, closely associated with the advanced retinopathy, independent of the traditional CVD risk factors, suggesting that patients with diabetes were more likely to develop a pattern of LV concentric remodeling [31] as the degree of retinopathy worsened [32]. To this point, the findings of our study were consistent with the previous research [33]. As reported, the myocardial structural changes of increased LV wall thickness are described as causes of diastolic dysfunction [34], which is featured in compromised LV relaxation and passive filling function. Hiramatsu et al. [35] further investigated that LV filling abnormalities were more intense among patients with DR, compared to those without retinopathy. Moreover, recent clinical studies have also shown that proliferative retinopathy has more advantages in predicting cardiovascular events compared to mild retinopathy [36]. These findings indirectly support our results. Thus, it is more reasonable to assume that retinopathy may represent extensive systemic microcirculation disorders, leading to an increased heart burden and impairing cardiac performance.

### 4.3. Retinopathy and Electrocardiogram

Our findings found that myocardial conduction function was significantly worse in patients with advanced retinopathy than in those without DR and that there was a strong correlation between retinopathy and abnormal electrocardiogram. It could be observed that patients with advanced retinopathy had a significantly increased heart rate and prolonged PR interval and QT interval over those without DR. More recent studies [37, 38] reported that prolonged PR interval and QT interval were associated with vascular endothelial disorders and were risk markers for vascular injury. It not only could suggest cardiac conduction dysfunction but also indirectly reflects the insufficiency of myocardial coronary blood supply [20, 39]. However, the specific mechanism of this combined phenotype is still unclear. The long-term chronic hyperglycemia may lead to oxidative stress response, inflammatory response, enhanced renin-angiotensin-aldosterone system (RAAS) activity, and vascular endothelial cell dysfunction, in which common pathogenesis could also be observed in the occurrence and development of DR. All pathologic mechanisms above could make a great contribution to the abnormal electrophysiology and conduction of cardiomyocytes [40, 41]. This may imply that either direct metabolic effects or contribution of retinopathy play a key role in the development of cardiac conduction. Furthermore, some investigators have demonstrated that DR and abnormal electrocardiograms were also closely related to diabetic autonomic neuropathy [42]. Although several previous studies have performed electrocardiogram arrhythmias in patients with diabetic micro- and macroangiopathy [43, 44], few data are available to analyze in detail the correlation between electrocardiogram abnormalities and DR stage. The present study suggested that diabetic retinopathy was closely related to PR and QT interval prolongation, implying that early attention should be paid to the cardiac electrical propagation process in DR patients to prevent cardiovascular events.

### 4.4. Clinical Implications

To our knowledge, this is the first study to evaluate in detail the relationship between the severity of DR, LV geometry, and electrocardiogram abnormalities. The findings of this research have the advantage of clinical applicability in that the emergence of advanced retinopathy could be used as an early warning for clinical monitoring of myocardial dysfunction. Our study suggests that diabetic patients suffering from severe retinopathy may be at greater risk of alterations in intracardiac impulse conduction and subclinical LV dysfunction. Notably, arterial hypertension is a widely recognized factor that causes cardiac alterations. In the present study, although blood pressure control was poor in patients with severe retinopathy, multivariate statistical analysis controlled for this confounding factor, and the findings remained stable and reliable. Therefore, it is reasonable to assume that retinopathy may indicate microvascular dysfunction not only in the retina but also in the heart and emphasize that the existence of retinopathy among diabetic patients, especially proliferative retinopathy, may require more aggressive cardiac evaluation.

### 4.5. Study Limitations

The limitations of this study should also be considered. Firstly, DR was graded based on a single fundoscopy, and patients without coronary artery disease did not undergo invasive coronary angiography. This problem could be solved using telemedicine in future studies, which allows recording multiple images of the fundus oculi. Secondly, the retrospective nature of this study limited our ability to evaluate the causal relationship between the severity of retinopathy and LV dysfunction. Finally, though we adjusted for the use of antihypertensive drugs and insulin in multivariate analysis, some oral antidiabetic drugs may have an independent impact on the remodeling and function of the heart, which needs to be taken into account when interpreting those results.

## 5. Conclusion

In conclusion, the findings of this study suggest that nearly all patients with T2DM had asymptomatic LV diastolic dysfunction, and the proliferative retinopathy was independently associated with LV hypertrophy. Interestingly, we also found that more severe retinopathy was significantly correlated with abnormalities of electrocardiogram in these subjects. Therefore, our findings show that the presence of retinopathy in patients with T2DM and preserved LVEF has clinical significance and suggests that clinicians should actively consider the further cardiac evaluation.

## Figures and Tables

**Table 1:** Comparison of clinical characteristics in T2DM patients stratified by retinopathy status.

Characteristics	Total (*n* = 790)	NDR (*n* = 475)	NPDR (*n* = 247)	PDR (*n* = 68)	*P* value
Male, *n* (%)	524 (66.3)	341 (71.8)	144 (58.3)^∗^	39 (57.4)^∗^	**<0.001**
Age (years)	52.87 ± 10.94	50.13 ± 10.76	57.60 ± 9.99^∗^	54.88 ± 9.26^∗^	**<0.001**
BMI (kg/m^2^)	25.52 ± 3.40	25.58 ± 3.37	25.46 ± 3.35	25.29 ± 3.77	0.761
Diabetes duration (years)	8.30 ± 6.35	6.05 ± 5.35	11.28 ± 6.06^∗^	13.16 ± 6.70^∗^^#^	**<0.001**
SBP (mmHg)	128.44 ± 17.62	124.67 ± 16.07	133.72 ± 18.81^∗^	135.65 ± 16.62^∗^	**<0.001**
DBP (mmHg)	79.47 ± 10.40	78.55 ± 10.62	80.68 ± 9.94^∗^	81.49 ± 9.74^∗^	**0.008**
Hypertension, *n* (%)	309 (39.1)	149 (31.4)	123 (49.8)^∗^	37 (54.4)^∗^	**<0.001**
HbA1c (%)	8.90 ± 2.04	8.84 ± 2.02	9.00 ± 2.04	8.86 ± 2.06	0.674
Total cholesterol (mmol/l)	4.19 ± 1.04	4.15 ± 0.97	4.19 ± 1.07	4.52 ± 1.31^∗^^#^	**0.021**
HDL-C (mmol/l)	1.02 ± 0.43	1.01 ± 0.47	1.01 ± 0.28	1.19 ± 0.53^∗^^#^	**0.006**
TG (mmol/l)	1.19 ± 1.36	1.99 ± 1.46	1.82 ± 1.20	1.71 ± 1.15	0.124
eGFR (ml/min/1.73 m^2^)	85.88 ± 18.73	91.29 ± 15.37	78.83 ± 20.37^∗^	73.87 ± 19.93^∗^^#^	**<0.001**
Uric acid, (*μ*mol/l)	275.53 ± 81.90	273.09 ± 79.76	276.26 ± 81.44	279.24 ± 92.98	0.825
Diabetic nephropathy, *n* (%)	195 (24.7)	70 (14.7)	86 (34.8)^∗^	39 (57.4)^∗^^#^	**<0.001**
Smoking history, *n* (%)	441 (60.5)	239 (53.8)	160 (70.8)^∗^	42 (71.2)^∗^	**<0.001**
Insulin use, *n* (%)	522 (66.0)	280 (58.9)	186 (75.3)^∗^	56 (82.4)^∗^	**<0.001**
ACEI/ARB use, *n* (%)	157 (20.0)	76 (16.0)	61 (24.7)^∗^	21 (30.9)^∗^	**0.001**

Note: data are expressed as means ± SD or *n* (%). Bold indicated value of *P* < 0.05. ^∗^*P* < 0.05 vs. NDR group. ^#^*P* < 0.05 vs. NPDR group. Abbreviations: T2DM: type 2 diabetes mellitus; NDR: no diabetic retinopathy; NPDR: nonproliferative diabetic retinopathy; PDR: proliferative diabetic retinopathy; BMI: body mass index; SBP: systolic blood pressure; DBP: diastolic blood pressure; HDL-C: high-density lipoprotein cholesterol; TG: triglyceride; eGFR: estimated glomerular filtration rate; ACEI: angiotensin converting enzyme inhibitor; ARB: angiotensin receptor blocker.

**Table 2:** Comparison of echocardiographic indices in T2DM patients stratified by retinopathy status.

Indices	Total (*n* = 790)	NDR (*n* = 475)	NPDR (*n* = 247)	PDR (*n* = 68)	*P* value
IVST (mm)	10.19 ± 1.20	10.00 ± 1.09	10.42 ± 1.21^∗^	10.66 ± 1.58^∗^	**<0.001**
LAD (mm)	35.57 ± 2.97	35.30 ± 2.77	35.78 ± 3.15^∗^	36.65 ± 3.34^∗^^#^	**0.001**
LV ESD (mm)	31.26 ± 2.69	31.54 ± 2.74	30.78 ± 2.48^∗^	31.03 ± 2.80	**0.001**
LV EDD (mm)	44.74 ± 3.48	45.13 ± 3.51	44.11 ± 3.34^∗^	44.26 ± 3.45	**<0.001**
LV EDV (ml)	74.42 ± 14.12	76.21 ± 14.31	71.59 ± 13.29^∗^	72.19 ± 13.92^∗^	**<0.001**
LV ESV (ml)	31.39 ± 7.10	32.16 ± 7.49	30.19 ± 6.17^∗^	30.41 ± 6.87	**0.001**
LV EF (%)	58.15 ± 2.94	58.17 ± 2.96	58.12 ± 2.91	58.06 ± 2.93	0.938
LVFS (%)	30.19 ± 2.48	30.20 ± 2.56	30.22 ± 2.39	30.06 ± 2.20	0.893
E velocity (cm/s)	55.50 ± 28.02	54.28 ± 29.26	56.73 ± 26.07	59.50 ± 25.75	0.258
A velocity (cm/s)	67.21 ± 34.40	61.61 ± 33.62	74.55 ± 34.17^∗^	79.40 ± 32.85^∗^	**<0.001**
E/A ratio	0.85 ± 0.29	0.90 ± 0.32	0.78 ± 0.19^∗^	0.76 ± 0.22^∗^	**<0.001**
Stroke volume (ml)	43.17 ± 7.91	44.07 ± 8.14	41.81 ± 7.35^∗^	41.79 ± 7.48^∗^	**<0.001**
E/A ratio < 1, *n* (%)	659 (84.6)	367 (78.3)	228 (94.2)^∗^	64 (94.1)^∗^	**<0.001**

Note: data are expressed as means ± SD or *n* (%). Bold indicated value of *P* < 0.05. ^∗^*P* < 0.05 vs. NDR group. ^#^*P* < 0.05 vs. NPDR group. Abbreviations: LV: left ventricular; IVST: interventricular septal thickness; LAD: left atrial diameter; LV ESD: LV end-systolic dimension; LV EDD: LV end-diastolic dimension; LV EDV: LV end-diastolic volume; LV ESV: LV end-systolic volume; LVEF: LV ejection fraction; LVFS: LV fractional shortening.

**Table 3:** Comparison of electrocardiogram indices in T2DM patients stratified by retinopathy status.

Indices	Total (*n* = 790)	NDR (*n* = 475)	NPDR (*n* = 247)	PDR (*n* = 68)	*P*
Heart rate (bpm)	74.66 ± 11.17	73.14 ± 10.65	76.51 ± 11.83^∗^	78.57 ± 10.44^∗^	**<0.001**
QRS (ms)	90.83 ± 16.81	91.51 ± 17.44	89.91 ± 13.86	89.44 ± 21.54	0.372
PR interval (ms)	161.97 ± 22.85	157.62 ± 20.09	169.62 ± 25.65^∗^	164.46 ± 22.76^∗^	**<0.001**
QT interval (ms)	363.39 ± 46.47	360.47 ± 42.44	367.94 ± 51.95^∗^	367.18 ± 39.14	0.087
QTc interval (ms)	402.47 ± 49.52	395.52 ± 45.85	411.78 ± 55.47^∗^	417.00 ± 43.01^∗^	**<0.001**
PR prolongation > 200 ms, *n* (%)	40 (5.1)	12 (2.5)	24 (9.7)^∗^	4 (5.9)^∗^	**<0.001**
QTc prolongation > 440 ms, *n* (%)	115 (14.6)	37 (7.8)	64 (25.9)^∗^	14 (20.6)^∗^	**<0.001**

Note: data are expressed as means ± SD or *n* (%). Bold indicated value of *P* < 0.05. ^∗^*P* < 0.05 vs. NDR group.

**Table 4:** The multiple linear regression analysis for clinical parameters with the electrocardiogram in T2DM patients.

Indices	Heart rate		PR interval		QTc interval	
	*β*	*P*	*β*	*P*	*β*	*P*
DR stage	1.593	**0.027**	4.666	**0.001**	8.807	**0.005**
Age	-0.032	0.485	0.386	**<0.001**	0.155	0.429
Male	-1.425	0.170	6.000	**0.004**	-14.335	**0.001**
DBP	0.152	**0.004**	-0.249	**0.018**	0.304	0.177
SBP	-0.011	0.743	0.164	**0.018**	-0.168	0.254
Hypertension	0.926	0.425	1.514	0.519	10.226	**0.041**
Total cholesterol	0.881	**0.031**	-0.079	0.924	4.881	**0.006**
HDL-C	-0.562	0.558	-1.882	0.333	-8.600	**0.038**
Diabetic nephropathy	2.574	**0.015**	3.252	0.129	1.398	0.759
Smoking history	0.162	0.871	0.644	0.748	-4.715	0.270
Diabetes duration	-0.004	0.959	-0.299	0.069	-0.068	0.846
ACEI/ARB use	0.948	0.455	0.004	0.912	-5.423	0.322
Insulin use	1.789	**0.044**	3.737	**0.038**	11.076	**0.004**

Note: bold indicated value of *p* < 0.05.

**Table 5:** Associated echocardiography parameters of DR in T2DM patients by univariable and age- and sex-adjusted logistic regression models.

	Univariable				Age- and sex-adjusted			
	*P*_NDR vs.NPDR_	OR (95% CI)	*P*_NDR vs.PDR_	OR (95% CI)	*P*_NDR vs.NPDR_	OR (95% CI)	*P*_NDR vs.PDR_	OR (95% CI)
IVST	**<0.001** ^∗^	1.36 (1.12-1.56)	**<0.001** ^∗^	1.56 (1.26-1.90)	**0.001** ^∗^	1.27 (1.10-1.47)	**<0.001** ^∗^	1.56 (1.27-1.91)
LAD	**0.038** ^∗^	1.06 (1.01-1.12)	**<0.001** ^∗^	1.16 (1.07-1.26)	0.083	1.05 (0.99-1.11)	**0.001** ^∗^	1.61 (1.07-1.26)
LV ESD	**<0.001** ^∗^	0.90 (0.85-0.95)	0.145	0.93 (0.85-1.03)	**0.030** ^∗^	0.93 (0.87-0.99)	0.474	0.96 (0.87-1.07)
LV EDD	**<0.001** ^∗^	0.92 (0.88-0.96)	0.054	0.93 (0.86-1.00)	**0.032** ^∗^	0.95 (0.90-0.99)	0.251	0.96 (0.88-1.03)
EDV	**<0.001** ^∗^	0.98 (0.96-0.99)	**0.030** ^∗^	0.98 (0.96-0.99)	0.053	0.99 (0.98-1.00)	0.284	0.99 (0.97-1.01)
ESV	**<0.001** ^∗^	0.96 (0.94-0.98)	0.064	0.96 (0.93-1.00)	0.141	0.98 (0.96-1.01)	0.408	0.98 (0.94-1.02)
LVEF	0.804	0.99 (0.94-1.05)	0.762	0.99 (0.90-1.08)	-	-	-	-
LVFS	0.907	1.00 (0.94-1.07)	0.666	0.98 (0.87-1.09)	-	-	-	-
Stroke volume	**<0.001** ^∗^	0.96 (0.94-0.98)	**0.028** ^∗^	0.96 (0.93-0.99)	0.154	0.98 (0.96-1.01)	0.263	0.98 (0.95-1.02)
E velocity	0.273	1.00 (0.99-1.01)	0.157	1.01 (0.99-1.02)	-	-	-	-
A velocity	**<0.001** ^∗^	1.01 (1.01-1.02)	**<0.001** ^∗^	1.02 (1.01-1.03)	0.125	1.00 (0.99-1.00)	**0.005** ^∗^	1.01 (1.00-1.02)
E/A ratio	**<0.001** ^∗^	0.16 (0.08-0.32)	**0.001** ^∗^	0.11 (0.03-0.41)	0.213	0.60 (0.27-1.34)	**0.027** ^∗^	0.19 (0.04-0.83)
E/A < 1 (yes vs. no)	**<0.001** ^∗^	4.53 (2.53-8.11)	**0.005** ^∗^	4.45 (1.58-12.50)	0.094	1.75 (0.91-3.35)	0.068	2.86 (0.93-8.84)

Note: data are expressed as OR (95% CI). “-” means variable not included. Bold indicated value of ^∗^*P* < 0.05. Abbreviations as Table 2.

**Table 6:** Associated echocardiography parameters of DR in T2DM patients by multivariable logistic regression models.

	Multivariable			
	*P*_NDR vs.NPDR_	OR (95% CI)	*P*_NDR vs.PDR_	OR (95% CI)
IVST	0.576	1.05 (0.88-1.26)	**0.026** ^∗^	1.35 (1.04-1.75)
LAD	0.503	1.02 (0.96-1.09)	0.098	1.09 (0.98-1.20)
LV ESD	**0.007** ^∗^	0.90 (0.84-0.97)	0.286	0.94 (0.83-1.06)
LV EDD	**0.012** ^∗^	0.93 (0.88-0.98)	0.160	0.93 (0.85-1.03)
EDV	**0.040** ^∗^	0.98 (0.97-1.00)	0.175	0.98 (0.96-1.01)
ESV	**0.049**	0.97 (0.94-1.00)	0.233	0.97 (0.92-1.02)
Stroke volume	0.112	0.98 (0.95-1.01)	0.245	0.98 (0.94-1.02)
A velocity	0.836	1.00 (0.99-1.01)	0.228	1.01 (1.00-1.02)
E/A ratio	0.580	0.78 (0.32-1.88)	0.284	0.40 (0.08-2.13)
E/A < 1 (yes vs. no)	0.183	1.63 (0.80-3.32)	0.181	2.64 (0.64-10.93)

Note: data are expressed as OR (95% CI). Bold indicated value of *P* < 0.05. ^∗^*P* < 0.05. Multivariable logistic regression model adjusted for age, gender, systolic and diastolic blood pressure, hypertension, total cholesterol, HDL-C, diabetic nephropathy, smoking history, duration of diabetes, ACEI/ARB use, and insulin use. Abbreviations as Table 2.

## Data Availability

The datasets used and/or analyzed during the current study are available from the corresponding authors upon reasonable request.
